# Preoperative prediction of early recurrence in resectable pancreatic cancer integrating clinical, radiologic, and CT radiomics features

**DOI:** 10.1186/s40644-024-00653-3

**Published:** 2024-01-08

**Authors:** Jeong Hyun Lee, Jaeseung Shin, Ji Hye Min, Woo Kyoung Jeong, Honsoul Kim, Seo-Youn Choi, Jisun Lee, Sungjun Hong, Kyunga Kim

**Affiliations:** 1 Department of Radiology and Center for Imaging Science, Samsung Medical Center, Sungkyunkwan University School of Medicine, 81 Irwon-ro Gangnam-gu, Seoul, 06351 Republic of Korea; 2 Department of Radiology, Soonchunhyang University Bucheon Hospital, Soonchunhyang University College of Medicine, Bucheon, Republic of Korea; 3 Department of Radiology, College of Medicine, Chungbuk National University, Chungbuk National University Hospital, Cheongju, Republic of Korea; 4 Department of Digital Health, Samsung Advanced Institute of Health Sciences and Technology (SAIHST), Sungkyunkwan University, Seoul, Republic of Korea; 5 Biomedical Statistics Center, Research Institute for Future Medicine, Samsung Medical Center, Seoul, Republic of Korea

**Keywords:** Pancreatic cancer, Prognosis, Radiomics, Tomography, X-ray computed, Machine learning

## Abstract

**Objectives:**

To use clinical, radiographic, and CT radiomics features to develop and validate a preoperative prediction model for the early recurrence of pancreatic cancer.

**Methods:**

We retrospectively analyzed 190 patients (150 and 40 in the development and test cohort from different centers) with pancreatic cancer who underwent pancreatectomy between January 2018 and June 2021. Radiomics, clinical-radiologic (CR), and clinical-radiologic-radiomics (CRR) models were developed for the prediction of recurrence within 12 months after surgery. Performance was evaluated using the area under the curve (AUC), Brier score, sensitivity, and specificity.

**Results:**

Early recurrence occurred in 36.7% and 42.5% of the development and test cohorts, respectively (*P* = 0.62). The features for the CR model included carbohydrate antigen 19-9 > 500 U/mL (odds ratio [OR], 3.60; *P* = 0.01), abutment to the portal and/or superior mesenteric vein (OR, 2.54; *P* = 0.054), and adjacent organ invasion (OR, 2.91; *P* = 0.03). The CRR model demonstrated significantly higher AUCs than the radiomics model in the internal (0.77 vs. 0.73; *P* = 0.048) and external (0.83 vs. 0.69; *P* = 0.038) validations. Although we found no significant difference between AUCs of the CR and CRR models (0.83 vs. 0.76; *P* = 0.17), CRR models showed more balanced sensitivity and specificity (0.65 and 0.87) than CR model (0.41 and 0.91) in the test cohort.

**Conclusions:**

The CRR model outperformed the radiomics and CR models in predicting the early recurrence of pancreatic cancer, providing valuable information for risk stratification and treatment guidance.

**Supplementary Information:**

The online version contains supplementary material available at 10.1186/s40644-024-00653-3.

## Introduction

Pancreatic cancer is a highly aggressive malignancy, which is challenging to treat, and has a 5-year survival rate of only 12% [1]. The poor prognosis of pancreatic cancer is primarily attributed to the high incidence of early postoperative recurrence, which up to 80% of the patients experience within 12 months after surgery [2]. Patients who experience early recurrence after surgical resection are unlikely to benefit from upfront surgery. Therefore, identifying patients at a high risk of early tumor recurrence before surgery may aid in selecting optimal treatment plan. Although several studies have developed preoperative predictive models based on clinical and radiological data, such as tumor size, lymphadenopathy, tumor differentiation, serum carbohydrate antigen 19-9 (CA19-9), and vascular abutment [3–5], reliable biomarkers indicative of the early recurrence of resectable pancreatic cancer are still lacking.

Radiomics is a subfield of radiology that involves the extraction and analysis of a large number of quantitative features from medical images [6]. As radiomics models have been applied to a variety of organ systems, these models are being developed in attempt to diagnose and determine the prognosis of pancreatic cancer [7–11]. These models, however, suffer from limitations, such as insufficient validation, small sample size, and the inability to capture information outside the tumor [7–11].

In the present multicenter study, we hypothesized that the combination of clinical, radiologic, and CT radiomics features would yield a more accurate prediction of early recurrence than clinical and/or radiologic features. We aimed to develop and validate a model, which integrated clinical, radiologic, and CT radiomics features, for the preoperative prediction of the early recurrence of pancreatic cancer.

## Methods

The present multicenter study was approved by the institutional review boards of the three tertiary referral centers (Samsung Medical Center IRB No: 2022-11-121, Soon Chun Hyang University Hospital Bucheon IRB No: 2023-02-024, Chungbuk National University IRB No.: 2023-02-020-001), and the requirement for informed consent was waived due to the retrospective study design. Our study adhered to the Transparent Reporting of a multivariable prediction model for Individual Prognosis Or Diagnosis guidelines [12].

### Patients

**Fig. 1:**
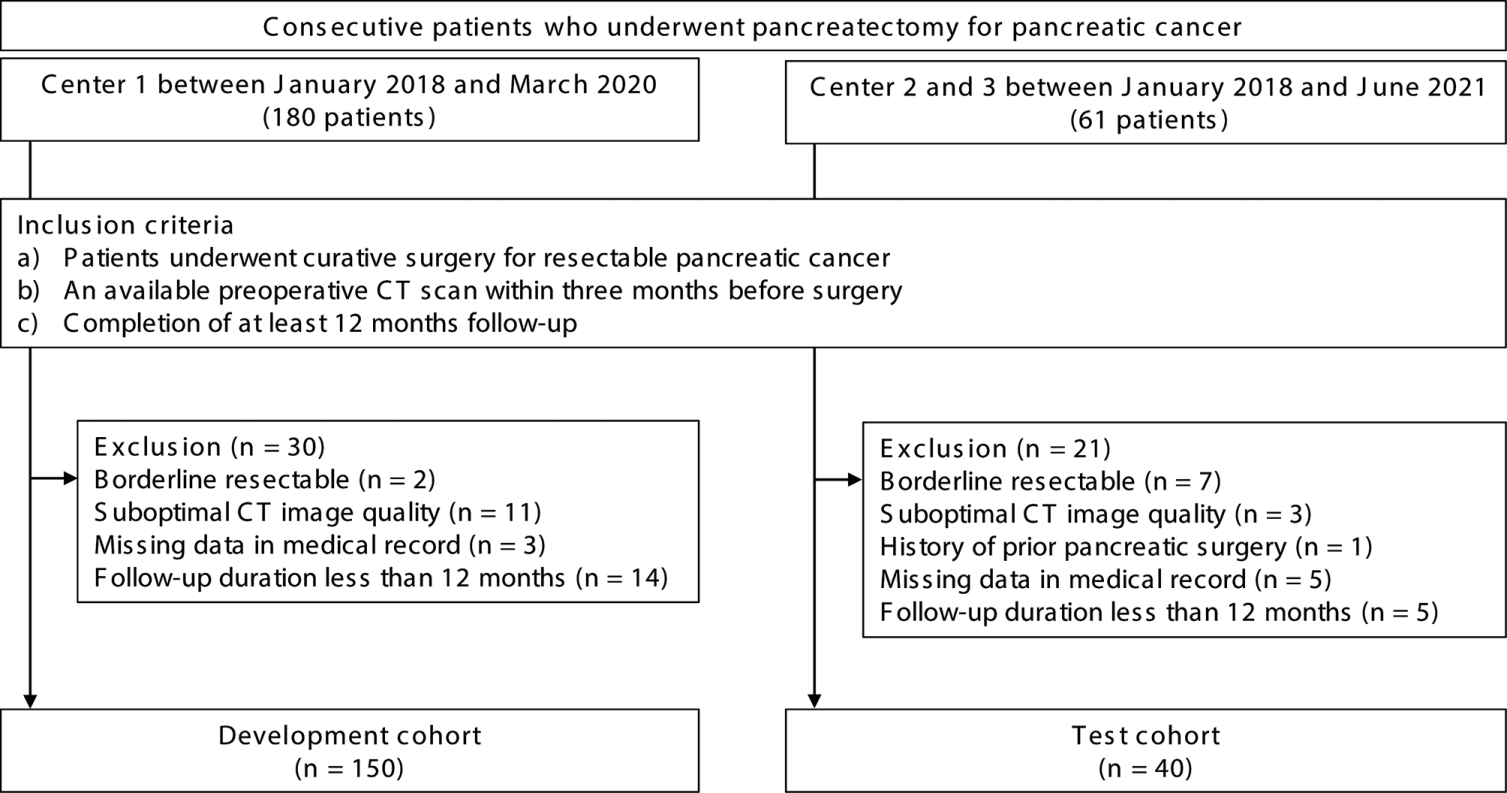
Flowchart of the study population

We searched the electronic databases of three hospitals for consecutive patients with pancreatic cancer who underwent pancreatectomy between January 2018 and June 2021. The inclusion criteria were as follows: (a) curative surgery for resectable pancreatic cancer as determined by a multidisciplinary team discussion among surgeons, oncologists, and radiologists in line with the National Comprehensive Cancer Network (NCCN) guidelines for pancreatic cancer version 2.2021 [13], (b) availability of a preoperative CT scan within 3 months of surgery, and (c) a follow-up period ≥ 12 months. The exclusion criteria were as follows: (a) borderline resectable pancreatic cancer according to the NCCN guidelines for pancreatic cancer version 2.2021, (b) suboptimal CT image quality, (c) history of prior pancreatic surgery, and (d) missing clinical data. The three centers included the following: (center 1) Samsung Medical Center, Seoul, South Korea formed the development cohort and (center 2) Soonchunhyang University College of Medicine, Bucheon Hospital, Bucheon, South Korea and (center 3) Chungbuk National University Hospital, Cheongju, South Korea, formed the test cohort. As detailed in Fig. 1, a total of 150 and 40 patients were included in the development and test cohorts, respectively.

### Clinical and pathological data

Clinical characteristics, such as age, sex, and preoperative serum CA19-9 levels obtained within one month before surgery, were collected from electronic medical records. Pathological results, including tumor size, tumor differentiation, T and N stages according to the American Joint Committee on Cancer tumor-node-metastasis 8th edition, and resection margin status, were also documented. Recurrence was defined as the presence of radiologic evidence of recurrent disease either at or adjacent to the surgical bed, including the remnant pancreas and locoregional nodes, or as evidence of recurrence detected outside these areas [14].

### CT acquisition and evaluation

Multidetector contrast-enhanced CT examinations were performed by each institution’s protocols. All CT examinations included the portal venous phase, by scanning the patient 70–80 s after the initiation of the contrast injection. The details of CT imaging techniques are summarized in Table S1.

Two board-certified abdominal radiologists (J.S. and J.H.L., with 10 and 9 years of experience, respectively), both blinded to the patients’ clinicopathological information other than the diagnosis of pancreatic cancer, independently reviewed the CT images. After their initial review, they reevaluated the images together to reach a consensus. They evaluated the following radiologic features: tumor location (pancreatic head/neck vs. body/tail), tumor abutment to the portal vein (PV) and/or superior mesenteric vein (SMV), peripancreatic tumor infiltration, adjacent organ invasion, lymph node enlargement, findings of obstructive pancreatitis, upstream parenchymal atrophy, and dilatation of the main pancreatic duct [4, 15–18]. Detailed definitions of the imaging features are provided in Appendix E1.

### Outcome measurement

Early recurrence was defined as recurrence of pancreatic cancer within the 12 months after surgery [2, 4]. After surgery, patients were followed up according to each institution’s protocol, through October 2022. In general, the patients’ follow-up included a clinical assessment, serum tumor marker evaluation, and CT or MRI every 3–6 months and at any time when clinically indicated.

### Development and validation of predictive models for the early recurrence of pancreatic cancer

**Fig. 2:**
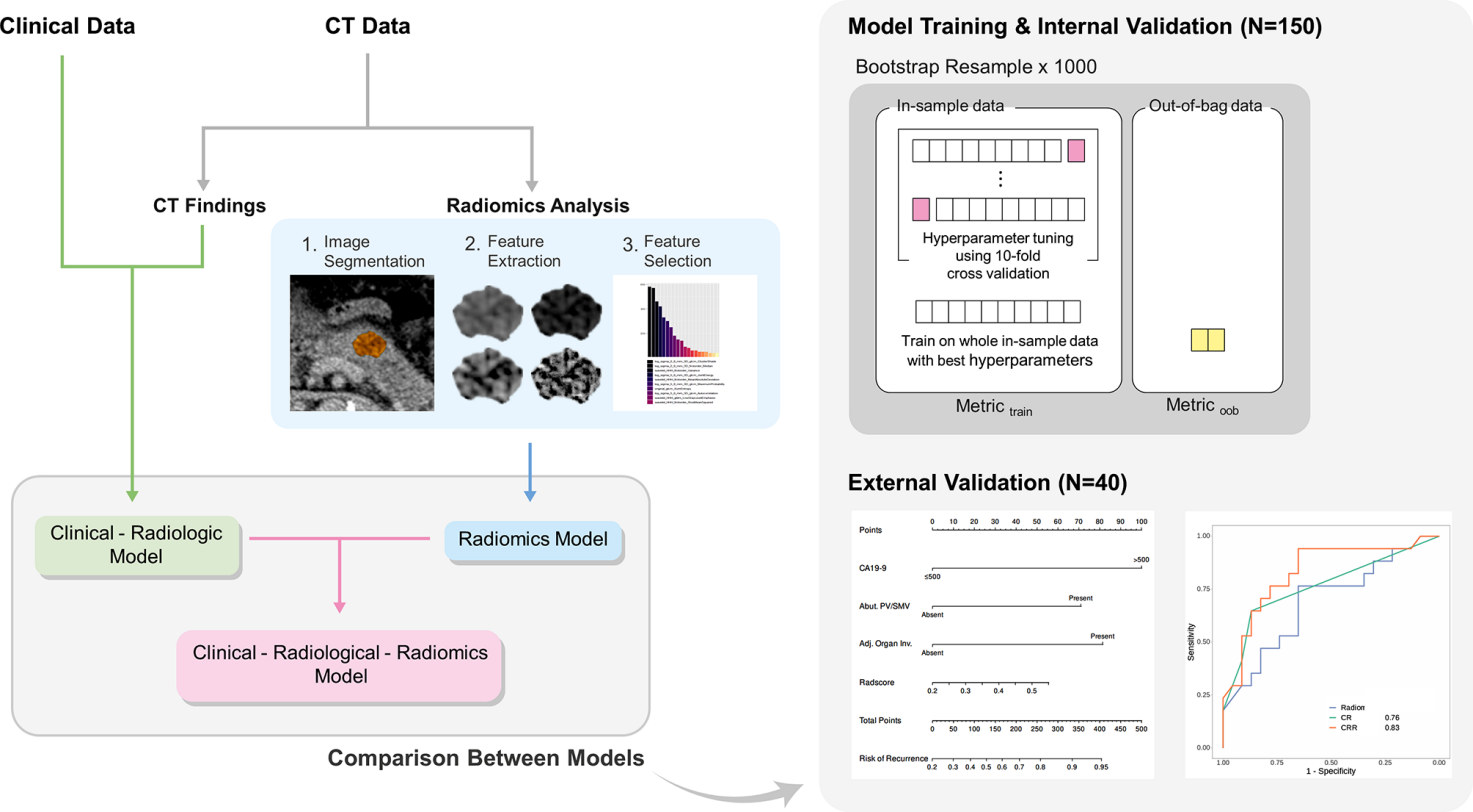
Training and validation flowchart

We developed the three models for the preoperative prediction of the early recurrence of pancreatic cancer in the development cohort (Fig. 2): (1) the radiomics model, based on CT radiomics features only; (2) clinical-radiologic (CR) model, based on clinical and radiologic features; and (3) the clinical-radiologic-radiomics (CRR) model, which combined the CR and radiomics models. The performance of each model was then validated externally using the test cohort.

### Segmentation

A radiologist (J.H.M., 13 years of experience in abdominal radiology), blinded to the patients’ clinical data except for pancreatic cancer diagnosis, manually segmented the pancreatic masses in both cohorts on the portal venous phase CT images by tracing along the tumor margins to create a volume of interest (VOI), using commercial software (Aview, version 1.0.38.6; Coreline Soft). To verify the feature stability of the interobserver variance of segmentation, another radiologist (J.H.L.) performed tumor segmentation in 20 randomly selected cases from the development cohort.

### Radiomics feature extraction and selection

Radiomics feature extraction was performed using the VOI of each tumor with PyRadiomics software (version 3.0.1; https://www.radiomics.io/pyradiomics.html) [19], in compliance with the Imaging Biomarker Standardization Initiative [20]. The original, Laplacian of the Gaussian (LoG)-filtered (sigma values of 2.0, 3.0, 4.0, and 5.0), and wavelet-filtered images were utilized to extract first-order, shape, and higher-order features. A total of 572 features were extracted, including 9 shape, 14 first-order, and 549 higher-order and filtered features.

Using data from the 20 cases, which were segmented independently by the aforementioned radiologists, the intraclass correlation coefficient (ICC) was calculated for all extracted features. Features with an interobserver ICC value > 0.75 were selected for subsequent process. Radiomics features were then selected using the exhaustive variant of the minimum redundancy maximum relevance ensemble [21], the top five features were selected for the radiomics model, based on the selection frequency.

### Development and selection of radiomics model

The process used for developing the radiomics model is illustrated in Fig. 2. The following three candidate algorithms were considered: logistic regression with L2 regularization (ridge regression); random forest; and Light Gradient Boosting Machine (LightGBM) [22, 23]. To compare the performance of the models, bootstrap resampling was utilized to create 1,000 bootstrap samples. For random forest and LightGBM, a 10-fold cross-validation was performed for each bootstrap sample. The set of hyperparameters that produced the best mean area under the receiver operating characteristic curve (AUC) was determined by cross-validation and was used to train the bootstrap sample [24]. Among the three models developed, the model with the highest AUC was selected as the final predictive model. The probability output from the final radiomics model was designated as the “Radscore”.

### Development of the CR model

To develop the CR model, univariable logistic regression was performed for each clinical and radiologic feature, and features with a *P*-value < 0.05 underwent multivariable logistic regression with backward selection using the Akaike information criterion. Features that survived backward selection were used to build the final CR model.

### Development of the CRR model

The CRR model was constructed by combining the predictors from the CR model and Radscore from the radiomics model using ridge regression. The CRR model was presented as a nomogram to calculate the risk of early pancreatic cancer recurrence.

### Statistical analysis

Comparisons between the development and test cohorts were done using chi-squared or the Fisher’s exact test for categorical variables, and student’s t-test or the Mann–Whitney U test for continuous variables. Interobserver agreement for the CT imaging features was evaluated using the kappa test as follows: poor, ≤0.20; fair, 0.21–0.40; moderate, 0.41–0.60; good, 0.61–0.80; and excellent, 0.81–0.99.

The performance of the models was evaluated using the AUC, Brier score, accuracy, sensitivity, and specificity. The Brier score is a measure of prediction accuracy, with lower scores indicating higher accuracy and 0 indicating a perfect prediction [25]. The thresholds for each model were set using the generalized threshold shifting technique, an algorithm-agnostic method for finding the optimal threshold for prediction by evaluating multiple subsets of the training data [26]. Internal validation was performed using 1,000-fold bootstrap samples with 0.632 + method for all metrics [27, 28] and with the percentile bootstrap method for 95% confidence interval (CI) [29].

In the development cohort, pairwise comparisons (radiomics vs. CRR and CR vs. CRR) of the AUCs were performed, and two-sided *P*-values were calculated by inverting the corresponding CIs [30, 31]. In the test cohort, pairwise AUC comparisons were performed using the Delong test. Statistical significance was set at a *P*-value < 0.05. The software packages used are listed in Appendix E2.

## Results

### Patients

The clinical and CT radiologic features, as well as the postoperative data, are summarized in Table 1. The development and test cohorts included a total of 150 (mean age, 66.3 years ± standard deviation [SD], 9.3; 69 males) and 40 (mean age, 69.3 years ± SD, 8.9; 18 males) patients, respectively. During the follow-up period, early recurrence occurred in 36.7% (55/150) and 42.5% (17/40) of the patients in the development and test cohort, respectively (*P* = 0.62). There were no significant differences in the demographic features, CA19-9 levels, CT radiologic features, or pathological tumor stages between the two cohorts, although the development cohort had a smaller tumor size (mean 2.6 vs. 2.8 cm; *P* = 0.048) and a lower proportion of head/neck tumors (34.7 vs. 60.0%; *P* = 0.01) than the test cohort. The proportion of patients who received adjuvant therapy showed a borderline difference between the two cohorts (70.7 vs. 87.5%; *P* = 0.050).

**Table 1:** Characteristics of development and test cohorts

	Development cohort (*n* = 150)	Test cohort (*n* = 40)	P-value
**Outcome**			
Recurrence within 1 year	55 (36.7)	17 (42.5)	0.62
**Pre-operative clinical features**			
Age, years (mean ± SD)	66.3 ± 9.3	69.3 ± 8.9	0.06
Sex			0.68
Male	69 (46.0)	18 (45.0)	
Female	81 (54.0)	22 (55.0)	
CA19-9 (mean ± SD)	551.0 ± 1,291.6	176.6 ± 320.4	0.07
**CT radiologic features**			
Tumor location			< 0.01
Head/neck	52 (34.7)	24 (60.0)	
Body/tail	98 (65.3)	16 (40.0)	
Abutment to the PV and/or SMV	27 (18.0)	6 (15.0)	0.83
Peripancreatic infiltration	72 (48.0)	19 (47.5)	> 0.99
Adjacent organ invasion	27 (18.0)	5 (12.5)	0.56
Enlarged lymph node	18 (12.0)	6 (15.0)	0.81
Obstructive pancreatitis	48 (32.0)	10 (25.0)	0.51
Upstream parenchymal atrophy	42 (28.0)	11 (27.5)	> 0.99
Main pancreatic duct dilatation	104 (69.3)	26 (65.0)	0.74
**Post-operative features**			
Tumor size, cm	2.6 ± 0.8	2.8 ± 0.7	0.048
Tumor differentiation			0.76
G1	7 (4.7)	2 (5.0)	
G2	117 (78.0)	33 (82.5)	
G3	26 (17.3)	5 (12.5)	
T stage			0.86
Tis	1 (0.7)	0 (0.0)	
T1	31 (20.7)	7 (17.5)	
T2	118 (78.7)	33 (82.5)	
N stage			0.93
N0	73 (48.7)	21 (52.5)	
N1	65 (43.3)	16 (40.0)	
N2	12 (8.0)	3 (7.5)	
Resection margin			0.39
R0	120 (80.0)	35 (87.5)	
R1	30 (20.0)	5 (12.5)	
Adjuvant chemotherapy/CCRT	106 (70.7)	35 (87.5)	0.050

*Note*: Unless otherwise indicated, data represent the number of patients, and numbers in parentheses indicate percentages

CA 19-9, carbohydrate antigen 19-9; CCRT, concurrent chemoradiotherapy; PV, portal vein; SD, standard deviation; SMV, superior mesenteric vein

### Radiomics model

The ICCs for the radiomics features ranged 0.02–1.00, with a mean value of 0.81, and 418 features had an ICC > 0.75. The following top five features were subsequently selected: 3D_cluster_shade of LoG-filtered images (sigma = 2); 3D_first_order_median of LoG-filtered (sigma = 2); 3D_joint_energy of LoG-filtered (sigma = 5); mean_absolute_deviation of wavelet-filtered (HHH); and variance of wavelet-filtered (HHH). The mean AUCs (95% CI) for the random forest, logistic regression, and LightGBM were 0.73 (0.57–0.84), 0.64 (0.51–0.73), and 0.71 (0.54–0.84), respectively, and the mean Brier scores (95% CI) were 0.20 (0.16–0.25), 0.22 (0.20–0.27), and 0.32 (0.30–0.35), respectively. Based on these results, the random forest algorithm was selected for the final radiomics model.

### CR model

Table 2 shows the frequency of each clinical and radiologic feature selected, and the results of the logistic regression analysis. Multivariable analysis revealed the following three features, which are predictive of the risk of early pancreatic cancer recurrence: CA19-9 > 500 U/mL (odds ratio [OR], 3.60; 95% CI, 1.39–9.34; *P* < 0.01), abutment to the PV and/or SMV (OR, 2.54; 95% CI, 0.98–6.56; *P* = 0.054), and adjacent organ invasion (OR, 2.91; 95% CI, 1.11–7.62; *P* = 0.03). Other than lymph node enlargement, the radiologic features showed moderate-to-excellent interreader agreement (Table S2).

**Table 2:** Logistic regression analysis for predicting early recurrence of pancreatic cancer in the development cohort

Variables	Prevalence (%)	Univariable		Multivariable			
				Clinical-radiologic model		Clinical-radiologic-radiomics model	
		Odds ratio (95% CI)	P-value	Odds ratio (95% CI)	P-value	Odds ratio (95% CI)	P-value
CA19-9 > 500 U/mL	30 (20.0)	5.90 (2.46–14.17)	< 0.01	3.60 (1.39–9.34)	0.01	2.91 (1.44–6.41)	0.01
Abutment to the PV and/or SMV	27 (18.0)	3.13 (1.33–7.38)	< 0.01	2.54 (0.98–6.56)	0.054	2.14 (1.05–4.39)	0.04
Peripancreatic infiltration	72 (48.0)	1.51 (0.78–2.95)	0.22				
Adjacent organ invasion	27 (18.0)	3.80 (1.59–9.07)	< 0.01	2.91 (1.11–7.62)	0.03	2.39 (1.10–5.19)	0.03
Enlarged lymph node	18 (12.0)	1.11 (0.40–3.06)	0.84				
Obstructive pancreatitis	48 (32.0)	0.92 (0.45–1.89)	0.83				
Upstream parenchymal atrophy	42 (28.0)	0.52 (0.23–1.13)	0.10				
Main pancreatic duct dilatation	104 (69.3)	0.58 (0.28–1.18)	0.13				
Radscore	–	–	–	–	–	5.46 (2.94–9.28)	< 0.01

CA 19-9, carbohydrate antigen 19-9; CI, confidence interval; PV, portal vein; SMV, superior mesenteric vein

### CRR model

**Fig. 3:**
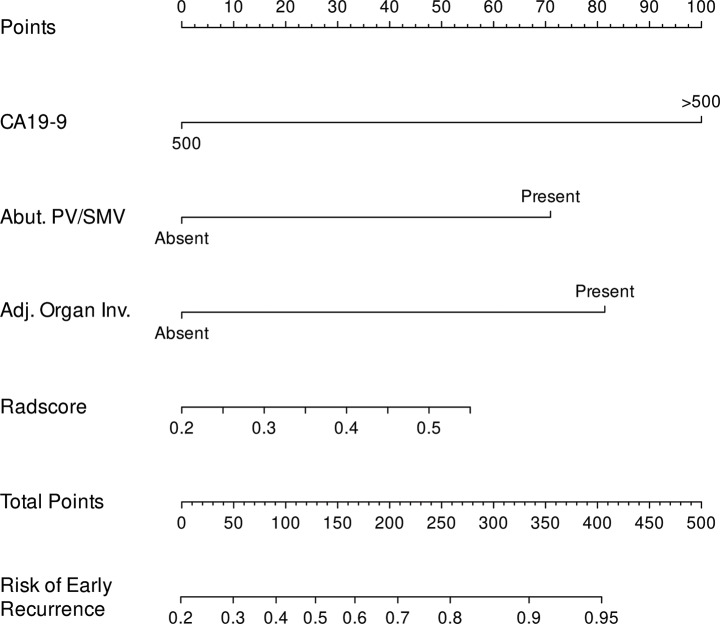
Nomogram of the clinical-radiologic-radiomics model for predicting the early recurrence of pancreatic cancer after surgery. The nomogram based on the clinical-radiologic-radiomics model for the prediction of early recurrence after pancreatectomy for resectable pancreatic cancer. To use the nomogram, the value for each variable is located on the corresponding axis and a line is drawn upward to determine the corresponding points value. The sum of these point values for all four predictive features is located on the total points axis, and a line is drawn downward to the survival axis to determine the likelihood of early cancer recurrence for an individual patient

**Fig. 4:**
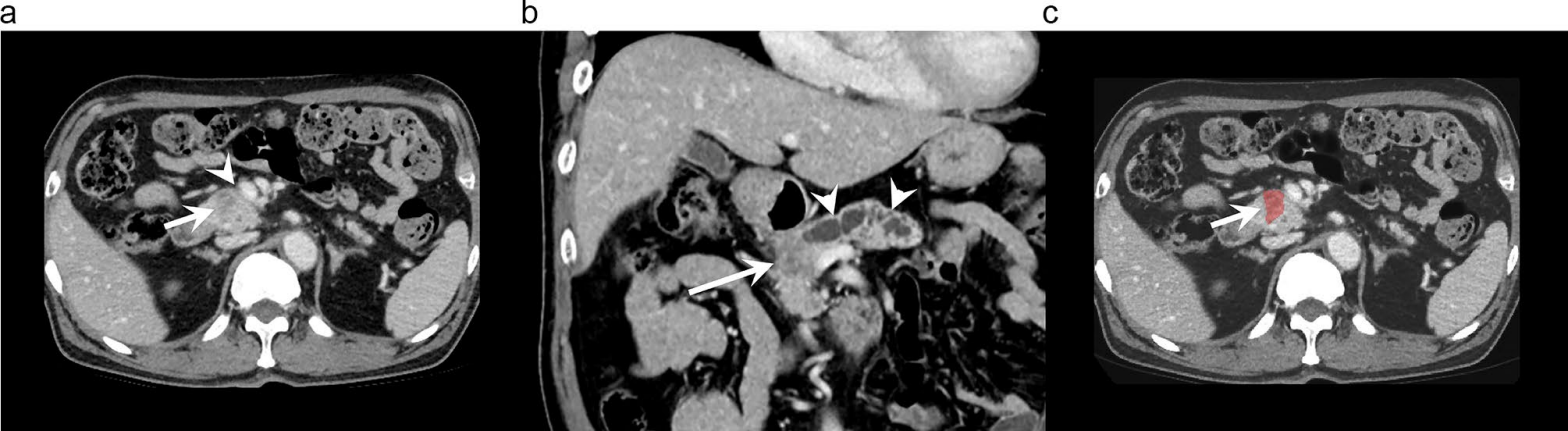
CT images of a 64-year-old male with pancreatic cancer. The patient’s preoperative CA19-9 level was 16.7 U/ml. (**a**) Axial portal venous phase CT image shows a 3.2 cm-sized pancreatic cancer (arrow) at the pancreas head. There was abutment of the tumor to the superior mesenteric vein (arrowhead), but no evidence of adjacent organ invasion. (**b**) Coronal portal venous phase shows the pancreatic cancer (arrow) with upstream main pancreatic duct dilatation (arrowheads). (**c**) Portal venous phase with segmentation overlay. An R0 resection was performed, with a pathological stage of T2N1. The CR model predicted non-early recurrence, while the CRR model predicted early recurrence. The patient experienced recurrence within 12 months, in the form of liver metastasis

Multivariable analysis of the combined model showed that a CA19-9 level > 500 U/mL (OR 2.91; 95% CI, 1.44–6.41; *P* = 0.01), abutment to the PV and/or SMV (OR, 2.14; 95% CI, 1.05–4.39; *P* = 0.04), and adjacent organ invasion (OR, 2.39; 95% CI, 1.10–5.19; *P* = 0.03) were predictive of early pancreatic cancer recurrence. Furthermore, the Radscore (OR, 5.46; 95% CI, 2.94–9.28; *P* < 0.01) was also found to be an independent predictor of early recurrence. The optimal threshold for the prediction of early recurrence was determined to be 0.41. The nomogram of the final CRR model is depicted in Fig. 3. CT images of representative cases with CR and CRR model discrepancy are shown in Fig. 4.

### Internal validation

In the development cohort, the AUCs (95% CI) of the radiomics, CR, and CRR models were 0.73 (0.57–0.84), 0.70 (0.60–0.77), and 0.77 (0.63–0.86), respectively (Table 3). The AUC of the CRR model was higher than that of the radiomics-only model (0.77 vs. 0.73; *P* = 0.048). Although the CCR model also had a higher AUC than the CR model, the difference was not statistically significant (0.70 vs. 0.77; *P* = 0.26). The sensitivities (95% CI) of the radiomics, CR, and CRR models were 0.75 (0.38–0.96), 0.50 (0.26–0.68), and 0.77 (0.49–0.96), respectively, while the specificities (95% CI) were 0.60 (0.46–0.84), 0.81 (0.66–0.93), and 0.61 (0.47–0.84), respectively.

**Table 3:** Model performance in predicting early recurrence of pancreatic cancer in the development and test cohorts

	AUC	P-value	Brier score	Accuracy	Sensitivity	Specificity
**Development cohort**						
Radiomics model	0.73 (0.57–0.84)	0.048^*^	0.20 (0.16–0.25)	0.64 (0.50–0.77)	0.75 (0.38–0.96)	0.60 (0.46–0.84)
CR model	0.70 (0.60–0.77)	0.26^*^	0.20 (0.18–0.24)	0.70 (0.61–0.76)	0.50 (0.26–0.68)	0.81 (0.66–0.93)
CRR model	0.77 (0.63–0.86)	–	0.19 (0.14–0.24)	0.66 (0.51–0.78)	0.77 (0.49–0.96)	0.61 (0.47–0.84)
**Test cohort**						
Radiomics model	0.69 (0.51–0.85)	0.038^†^	0.24 (0.22–0.25)	0.45 (0.43–0.50)	1.00 (1.00–1.00)	0.04 (0.00–0.13)
CR model	0.76 (0.56–0.83)	0.17^†^	0.21 (0.20–0.26)	0.70 (0.53–0.78)	0.41 (0.12–0.53)	0.91 (0.78–1.00)
CCR model	0.83 (0.65–0.94)	–	0.20 (0.18–0.24)	0.78 (0.60–0.85)	0.65 (0.29–0.77)	0.87 (0.70–1.00)

Data are presented as mean (95% CI)

^*^Pairwise comparison with CCR model using bootstrap

^†^Pairwise comparison with CCR model using Delong’s test

AUC, area under the receiver operating characteristic curve; CI, confidence interval; CR, clinical-radiologic; CRR, clinical-radiologic-radiomics

### External validation

**Fig. 5:**
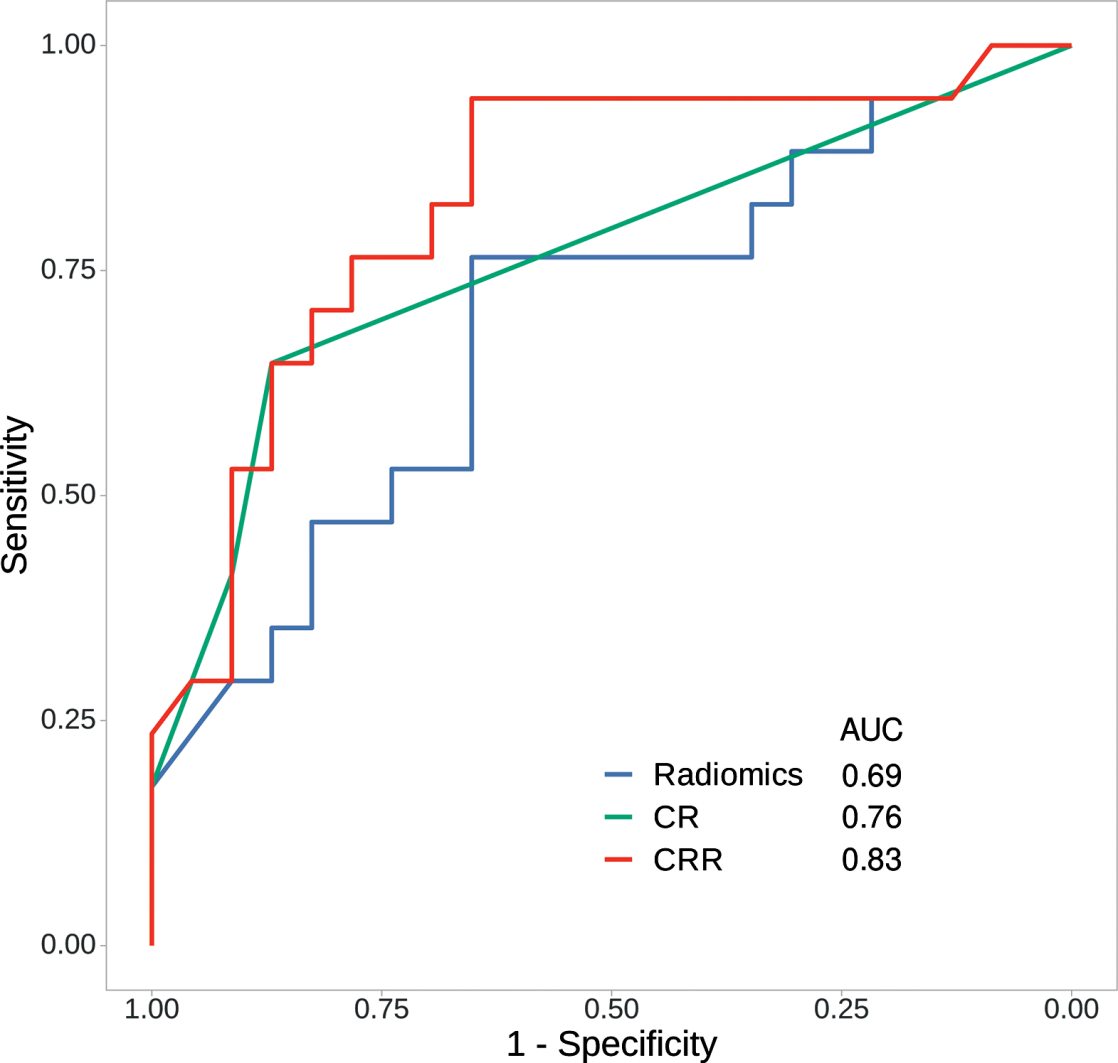
Receiver operating characteristic (ROC) curves of the radiomics, CR, and CRR models in the test cohort. The AUC of the CRR model in the test cohort was higher than that of the radiomics model (0.69 vs. 0.83; *P* = 0.038). AUC, area under the curve; CR, clinical-radiologic; CRR, clinical-radiologic-radiomics

In the test cohort, the AUC (95% CI) of each model was as follows (Table 3): 0.69 (0.51–0.85) for radiomics, 0.76 (0.56–0.83) for CR, and 0.83 (0.65–0.94) for CRR. When the CR model was combined with the radiomics model, the resulting CRR model showed a higher AUC value than the radiomics model alone (0.69 vs. 0.83; *P* = 0.038). The sensitivity (95% CI) of each model was as follows: 1.00 (1.00–1.00) for radiomics, 0.41 (0.12–0.53) for CR, and 0.65 (0.29–0.77) for CRR. The specificity (95% CI) of each model was as follows: 0.04 (0.00–0.13) for radiomics, 0.91 (0.78–1.00) for CR, and 0.87 (0.70–1.00) for CRR. Additionally, the increase in the Brier score for the radiomics model (0.04; 0.20 vs. 0.24) was greater than those of the CR (0.01; 0.20 vs. 0.21) and CRR (0.01; 0.19 vs. 0.20) models. The ROC curves for the three models are shown in Fig. 5.

## Discussion

We developed radiomics-, CR-, and CRR-based models for the prediction of the early recurrence of pancreatic cancer within 12 months after surgical resection. The CRR model demonstrated better performance than the radiomics model alone, with a better AUC in both the development and test cohorts. External validation supported the effectiveness of the CRR model, indicating that integrating radiomics with clinical and radiologic factors enhanced the predictive accuracy for the early recurrence of pancreatic cancer.

Patients at high risk of early recurrence of pancreatic cancer might not benefit from an immediate surgical resection, which can result in significant morbidity [32]. The accurate prediction of early recurrence, therefore, can guide treatment decisions, such as determining which patients may benefit from neoadjuvant chemotherapy [33–36]. Unfortunately, there is no widely accepted model for predicting the early recurrence of pancreatic cancer after curative resection. Previous models have limited clinical utility because they rely only on postoperative or pathological findings as predictive features. Predictions using those models, therefore, can only be made after surgery and as such are not beneficial to the decision to preclude upfront surgical resection [3, 37, 38]. CA19-9 level has been the most consistent and widely accepted prognostic factor [17, 39], but its performance is limited, as its ability to predict early recurrence was reported to have an AUC of 0.68 [17]. Another issue with CA19-9 levels as a predictive factor is that they are heavily influenced by obstructive jaundice [39, 40]. Meanwhile, radiologic features, such as tumor size, hypodensity during the portal venous phase, invasion of adjacent organs, and contact with the PV and/or SMV are associated with pancreatic cancer prognosis [4, 15, 16]; however, their utility is limited due to the moderate interobserver agreement [41].

Previous studies have explored the potential applications of radiomics-based models in determining the prognosis of pancreatic cancer [42–46]; however, this approach is unable to capture information outside of the tumor, whereas the CR model may provide valuable peritumoral information, such as PV and/or SMV abutment or invasion of adjacent organs. Accordingly, the radiomics model in the present study did not demonstrate sufficient predictive ability for the early recurrence of pancreatic cancer. A previous study attempted to include perilesional information by increasing the segmentation boundary; however, this approach could not take into account broader contextual information, such as adjacent organ invasion and vascular abutment, which were also demonstrated to be significant in the present study [42]. One promising approach for enhancing radiomics-based models involves providing additional information, such as radiologic features and clinical data, that cannot be obtained from a segmented image alone [47]. The results of the present study support this hypothesis, as the combined CRR model performed better than the radiomics-only model.

The present study compared several machine-learning algorithms in developing a radiomics-based prediction model. Random forest, a model built on the bootstrap aggregation algorithm, seeks to avoid overfitting by an ensemble of multiple decision trees trained on different subsets of training data, thereby offering robust performance and noise resistance [48]. The random forest algorithm showed the best performance in the present study, which is consistent with previous studies on various organs [49–51]; however, compared to the CR and CRR models, the random forest-based radiomics model exhibited a significant decrease in accuracy and specificity with the largest increase in the Brier score on external validation. Potential causes for this decreased performance include variations in CT scanners and protocols, as well as differences in patient and tumor characteristics across different cohorts [52]. Accordingly, while modern machine learning algorithms deliver impressive results, their performance may deteriorate significantly when applied to different clinical scenarios. Therefore, it is crucial to assess these models with various clinical settings and imaging protocols to ensure their efficacy in real world practice.

The present study had several limitations. First, due to the retrospective nature of the study, selection bias might have been introduced, despite efforts to minimize it. Second, our model did not account for postoperative factors that may affect recurrence, such as the resection margin status and the administration of adjuvant therapy. As we focused on the development of preoperative model for early recurrence to identify patients who may benefit from alternative treatments, those potential factors, which can be obtained after surgery, were not included in our model. Third, our cohorts exhibited a high average CA19-9 level, which was a significant variable in the final model. Therefore, additional research is needed to determine whether our model can be generalized to patients with normal CA 19-9 levels. Fourth, the development and testing cohorts were limited in size. Although bootstrapping was employed, a larger sample size would have produced more reliable results and potentially improved the radiomics model. Fifth, although interobserver variance in tumor segmentation was addressed using the ICC, more accurate automatic segmentation may decrease potential measurement errors. Unfortunately, automatic segmentation of the pancreas is not sufficiently reliable as of now.

## Conclusion

In conclusion, we have developed and compared the performance of radiomics, CR, and CRR models for predicting early recurrence in operable pancreatic cancer. The CRR model outperformed the radiomics and CR models in predicting the early recurrence of pancreatic cancer, providing valuable information for risk stratification and treatment guidance prior to surgical resection of pancreatic cancer.

### Electronic supplementary material

Below is the link to the electronic supplementary material.

Supplementary Tables and Appendix

## Data Availability

The data supporting the conclusions of this article is(are) available in the author’s github repository, [https://github.com/sigjhl/pdac_prognosis]. Our patient data is not included in the repository, but available upon reasonable request.

## Abbreviations

AUC: area under the curve
CA19-9: carbohydrate antigen 19-9
CI: confidence interval
CR: clinical-radiologic
CRR: clinical-radiologic-radiomics
CT: computed tomography
GBM: gradient boosting machine
ICC: intraclass correlation coefficient
LoG: Laplacian of the Gaussian
OR: odds ratio
PV: portal vein
SD: standard deviation
SMV: superior mesenteric vein
VOI: volume of interest
