# Supplementary material for: Preoperative prediction of early recurrence in resectable pancreatic cancer integrating clinical, radiologic, and CT radiomics features
Source: Cancer Imaging. 2024 Jan 8;24:6. doi: 10.1186/s40644-024-00653-3 (PMC10775464; doi:10.1186/s40644-024-00653-3)
Supplement: Supplementary file 1 — Supplementary Tables and Appendix [file 40644_2024_653_MOESM1_ESM.docx]

**Table S1.** CT imaging techniques

| **CT techniques** | **Development cohort**  **(n = 150)** | | **Test cohort**  **(n = 40)** | |
| --- | --- | --- | --- | --- |
| Tube voltage, n (%) |  |  |  |  |
| 80–110 kVp | 25 | (16.7) | 8 | (20.0) |
| 120 kVp | 121 | (80.7) | 30 | (75.0) |
| 130–140 kVp | 4 | (2.7) | 2 | (5.0) |
| Slice thickness, n (%) |  |  |  |  |
| 2–4 mm | 104 | (69.3) | 37 | (92.5) |
| 5 mm | 46 | (30.7) | 3 | (7.5) |
| CT vendors, n (%) |  |  |  |  |
| GE | 68 | (45.3) | 11 | (27.5) |
| Hitachi | 4 | (2.7) | 0 | (0) |
| Philips | 16 | (10.7) | 5 | (12.5) |
| Siemens | 38 | (25.3) | 22 | (55.0) |
| Toshiba | 24 | (16.0) | 2 | (5.0) |

**Table S2.** Inter-reader agreement of radiologic features

| **Variable** | **Kappa** | **95% CI** | **Interpretation** |
| --- | --- | --- | --- |
| Tumor location | 0.92 | 0.80–1.00 | Excellent agreement |
| Tumor abutment to the PV and/or SMV | 0.67 | 0.36–0.98 | Good agreement |
| Peripancreatic infiltration | 0.68 | 0.47–0.88 | Good agreement |
| Adjacent organ invasion | 0.75 | 0.52–0.98 | Good agreement |
| Lymph node enlargement | 0.20 | -0.21–0.81 | Poor agreement |
| Findings of obstructive pancreatitis | 0.60 | 0.35–0.86 | Moderate agreement |
| Upstream parenchymal atrophy | 0.95 | 0.86–1.00 | Excellent agreement |
| Dilatation of the main pancreatic duct | 0.85 | 0.69–1.00 | Excellent agreement |

CI, confidence interval; PV, portal vein; SMV, superior mesenteric vein

**Appendix E1.** Definition of CT imaging features

1. Tumor abutment to the portal vein (PV) and/or superior mesenteric vein (SMV): Solid soft tissue contact with the PV and/or SMV under 180°.
2. Peripancreatic infiltration: Hazy or stranding attenuation around the tumor.
3. Adjacent organ invasion: Invasion of the duodenum, stomach, common bile duct, spleen, or bowels.
4. Lymph node enlargement: Regional lymph nodes with short diameter >10 mm.
5. Obstructive pancreatitis: Findings of pancreatitis upstream to the tumor. The pancreatitis was defined as the presence of localized or diffuse enlargement of the pancreas, decreased and heterogeneous parenchymal enhancement, ill-defined parenchymal contours, and peripancreatic fat stranding, with or without peripancreatic fluid collection and parenchymal necrosis [1].
6. Upstream parenchymal atrophy: Disproportionate atrophic change of the pancreas parenchyma upstream to the tumor [2].
7. Dilatation of the main pancreatic duct: Dilatation of the pancreatic duct ≥ 2 mm when there is abrupt cut-off of the main pancreatic duct or when the pancreatic duct proximal to the cut-off site is wider than the pancreatic duct distal to the duct cut-off site [3-6].

**Appendix E2.** The packages used for statistical analysis

Feature selection in the radiomics and clinical-radiologic models was performed using the minimum redundancy maximum relevance ensemble (mRMRe) 2.1.2 and glmnet 4.1.4 packages on R 4.2.2 [7, 8]. All model training and hyperparameter tuning were performed using scikit-learn 1.2.0, and Optuna 3.0.3, in Python 3.10.6 [9, 10]. The calculation of .632+ metrics was adopted from the mlxtend package 0.21.0 [11].

**References**

1. Turkvatan A, Erden A, Turkoglu MA, Secil M, Yener O (2015) Imaging of acute pancreatitis and its complications. Part 1: acute pancreatitis. Diagn Interv Imaging 96:151-160

2. Yamao K, Takenaka M, Ishikawa R et al (2020) Partial Pancreatic Parenchymal Atrophy Is a New Specific Finding to Diagnose Small Pancreatic Cancer (≤10 mm) Including Carcinoma in Situ: Comparison with Localized Benign Main Pancreatic Duct Stenosis Patients. Diagnostics 10:445

3. Tanaka S, Nakaizumi A, Ioka T et al (2002) Main pancreatic duct dilatation: a sign of high risk for pancreatic cancer. Jpn J Clin Oncol 32:407-411

4. Tanaka S, Nakao M, Ioka T et al (2010) Slight dilatation of the main pancreatic duct and presence of pancreatic cysts as predictive signs of pancreatic cancer: a prospective study. Radiology 254:965-972

5. Yoon SH, Lee JM, Cho JY et al (2011) Small (</= 20 mm) pancreatic adenocarcinomas: analysis of enhancement patterns and secondary signs with multiphasic multidetector CT. Radiology 259:442-452

6. Johnston A, Serhal A, Lopes Vendrami C et al (2020) The abrupt pancreatic duct cutoff sign on MDCT and MRI. Abdominal Radiology 45:2476-2484

7. De Jay N, Papillon-Cavanagh S, Olsen C, El-Hachem N, Bontempi G, Haibe-Kains B (2013) mRMRe: an R package for parallelized mRMR ensemble feature selection. Bioinformatics 29:2365-2368

8. Friedman J, Hastie T, Tibshirani R (2010) Regularization paths for generalized linear models via coordinate descent. Journal of statistical software 33:1

9. Akiba T, Sano S, Yanase T, Ohta T, Koyama M. Optuna: A next-generation hyperparameter optimization framework. Proceedings of the 25th ACM SIGKDD international conference on knowledge discovery & data mining, 2019:2623-2631.

10. Pedregosa F, Varoquaux G, Gramfort A et al (2011) Scikit-learn: Machine learning in Python. Journal of Machine Learning Research 12:2825-2830

11. Raschka S (2018) MLxtend: Providing machine learning and data science utilities and extensions to Python’s scientific computing stack. Journal of Open Source Software 3:638
